# Hybridization among Three Native North American *Canis* Species in a Region of Natural Sympatry

**DOI:** 10.1371/journal.pone.0003333

**Published:** 2008-10-08

**Authors:** Frank Hailer, Jennifer A. Leonard

**Affiliations:** 1 Department of Evolutionary Biology, Evolutionary Biology Centre, Uppsala University, Uppsala, Sweden; 2 Center for Conservation and Evolutionary Genetics, National Zoological Park & National Museum of Natural History, Smithsonian Institution, Washington, D. C., United States of America; University of Utah, United States of America

## Abstract

**Background:**

Population densities of many species throughout the world are changing due to direct persecution as well as anthropogenic habitat modification. These changes may induce or increase the frequency of hybridization among taxa. If extensive, hybridization can threaten the genetic integrity or survival of endangered species. Three native species of the genus *Canis*, coyote (*C. latrans*), Mexican wolf (*C. lupus baileyi*) and red wolf (*C. rufus*), were historically sympatric in Texas, United States. Human impacts caused the latter two to go extinct in the wild, although they survived in captive breeding programs. Morphological data demonstrate historic reproductive isolation between all three taxa. While the red wolf population was impacted by introgressive hybridization with coyotes as it went extinct in the wild, the impact of hybridization on the Texas populations of the other species is not clear.

**Methodology/ Principal Findings:**

We surveyed variation at maternally and paternally inherited genetic markers (mitochondrial control region sequence and Y chromosome microsatellites) in coyotes from Texas, Mexican wolves and red wolves from the captive breeding programs, and a reference population of coyotes from outside the historic red wolf range. Levels of variation and phylogenetic analyses suggest that hybridization has occasionally taken place between all three species, but that the impact on the coyote population is very small.

**Conclusion/Significance:**

Our results demonstrate that the factors driving introgressive hybridization in sympatric Texan *Canis* are multiple and complex. Hybridization is not solely determined by body size or sex, and density-dependent effects do not fully explain the observed pattern either. No evidence of hybridization was identified in the Mexican wolf captive breeding program, but introgression appears to have had a greater impact on the captive red wolves.

## Introduction

Hybridization between animal species in the wild is revealed in an increasing number of studies [1]–[3]. In situations when one or both of the taxa involved is/are rare, Allee effects [4] can lead to a breakdown of prezygotic reproductive barriers and initiate genetic introgression [5]–[8]. A high frequency of hybridization events followed by backcrossing may lead to the formation of a hybrid swarm, and in the most extreme case, result in species replacement (e.g. [9]). Hybridization may have become more frequent in recent times due to population declines, translocation of species outside of their native range, and anthropogenic habitat modifications [1], [8]. This has important conservation implications.

There are multiple examples in the genus *Canis* where hybridization is a serious threat to the survival of an endangered species or population. For example, hybridization with domestic dogs (*C. familiaris*) threatens the Simian wolf (*C. simensis*) [10] and hybridization with coyotes (*C. latrans*) threatens the red wolf (*C. rufus*) [11]–[13]. Another case may be the Great Lakes area wolves (*C. lupus lycaon*) that have hybridized both with gray wolves (*C. lupus nubilus*) and coyotes [14]. In all these cases, there is a large disparity in population size between the hybridizing taxa, and the species that is rare is threatened by interbreeding with the common species. However, in other parts of the range of these same species, hybridization has not been observed [15]–[18] in spite of very disparate numbers. Some examples are the recently reintroduced population of gray wolves in Yellowstone National Park [19] and the naturally recolonizing wolves in the Rocky Mountains [17] that co-exist with large numbers of coyotes. This suggests that the conditions leading to hybridization in *Canis* are more complex than simple differences in abundance.

**Figure 1:**
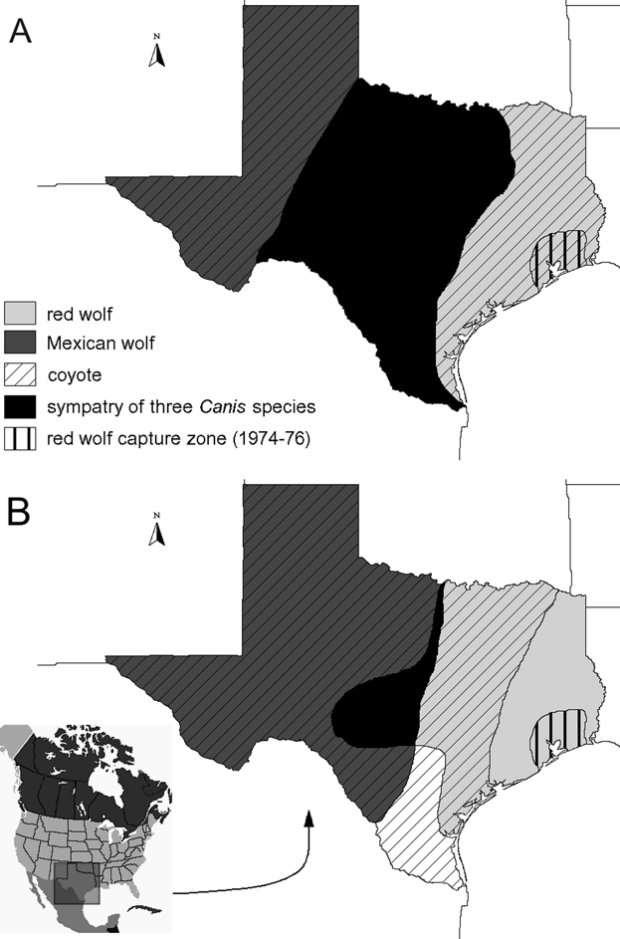
Historic distribution of three species of *Canis* in Texas. The region of historic sympatry is shown in black. Diagonal hatching denotes the coyote distribution, light gray shading that of red wolves, and dark gray shading that of Mexican wolves. The vertically striped region in southeastern Texas indicates where the founders of the red wolf captive breeding program were caught in 1974–76 [27]. *A:* Ca. 1700 C.E. distribution ranges following Carbyn [56] and Young & Goldman [21]. *B:* Ca. 1500 C.E. distribution ranges based on Nowak [20].

Texas is a region where three species of *Canis* historically occurred in sympatry (Fig. 1). Two of them, the Mexican wolf (*C. lupus baileyi*, a subspecies of gray wolf) and the red wolf, went extinct in the wild but were preserved in captive breeding programs. The third species is the coyote, which remains extant in the wild and is currently abundant. Historical levels of hybridization are unknown, but morphological data from historical specimens demonstrate that introgression, if it occurred, had not led to the formation of a hybrid swarm prior to recent human impacts [20]. However, population declines of the Mexican and red wolves during the 20th century could have resulted in an increased frequency of hybridization.

Hybridization with coyotes currently threatens the reintroduced red wolf population in North Carolina [13]. This threat is not new-the founders of the captive breeding program originated from a population known to have been impacted by hybridization with coyotes [11], [21]. Red wolf–coyote hybrids may have backcrossed into the coyote population as well as the red wolf population. If this was the case, then red wolf genetic material could still persist in the wild population of coyotes in Texas.

Mexican wolves were driven to extinction in the wild by many of the same causes that led to the decline of the red wolf [22]. Hybridization between Mexican wolves and the other *Canis* species is possible, implying that Mexican wolves may also have left a legacy of introgressed genetic material in the extant wild coyotes in Texas. Hybridization may also have affected the founders of the Mexican wolf captive breeding program.

Application of genetic markers can shed light on questions related to past hybridization events. However, alleles at commonly used nuclear markers such as autosomal microsatellites are often shared between closely related taxa see [23], [24], so inferences are to a large degree based on allele frequency differences. When populations go through bottlenecks, such as when the last few wild red wolves and Mexican wolves were captured to be founders of the captive breeding programs, they are subject to strong genetic drift. This drift may substantially alter the occurrence of alleles [25], posing a challenge to genetic inferences based on allelic frequencies. In these cases, haploid genetic markers, such as mitochondrial DNA (mtDNA) or Y chromosome markers, may be more informative [24]. These markers have a faster coalescence (due to a smaller effective population size), making taxon-specific alleles more prevalent. Further, since hybridization may be directional and sex-biased, separate analysis of both maternally and paternally inherited markers may yield important insights into the hybridization process.

Here we investigate the role of hybridization between three species of the genus *Canis* (O. Carnivora, Fam. Canidae) in North America, of which two went extinct in the wild due to human impact. We used maternally (mtDNA control region sequences) and paternally (Y chromosome microsatellites) inherited markers to analyze the coyote population from Texas, and to compare it to the red wolf, the Mexican wolf, and a population of coyotes from an area in Nebraska where historically only coyotes and gray wolves coexisted.

## Materials and Methods

### Samples

Tissue samples were collected from culled wild coyotes in Texas (*n* = 53), from western Texas (Andrews Co. *n* = 12) and southern Texas (Webb Co., *n* = 41). DNA samples were obtained from animals from the captive breeding programs of red wolves (*n* = 5 males studbook numbers 224, 387, 294, 352, 357; founders were caught in Texas; Fig. 1) and Mexican wolves (*n* = 16 males; McBride *n* = 5, Ghost Ranch *n* = 7 and Aragon *n* = 4 studbook numbers SB7, SB44, SB47, SB60, SB67, GRMLO36, GR1, GR3, GR5, GR91-22, GR91-42, GR91-43, A1, A3, A5, A6). In addition, previously published mitochondrial DNA data on historic [26] and recent [18] Mexican wolves, Texas coyotes [13], and historic [27], [28] and recent red wolves [18] were also included. In order to determine if levels of genetic variability at maternally and paternally inherited markers have changed dramatically due to hybridization in coyotes from Texas, we also obtained tissue samples from culled wild coyotes in Nebraska (*n* = 75), from an area where historically only coyotes and gray wolves coexisted. Finally, we gathered previously published Y chromosome data from gray wolves [29]–[32] for phylogenetic and diversity comparisons.

### Molecular methods

DNA was extracted using a standard phenol-chloroform extraction followed by alcohol precipitation [33]. The 5′ end of the mtDNA control region was amplified with primers ThrL 5′-GAA TTC CCC GGT CTT GTA AAC C-3′ and DLH-can 5′-CCT GAG GTA AGA ACC AGA TG-3′ from [34] as in [18]. Polymerase chain reaction (PCR) products were directly sequenced with BigDye terminator chemistry (Perkin-Elmer, Boston, Massachusetts) using the same primers as in the PCR. Sequences were run on an ABI automated sequencer 377 (Perkin-Elmer, Boston, Massachusetts) following the manufacturer's protocols and subsequently checked and aligned by eye using Sequencher version 4.6 (Gene Codes, Ann Arbor, USA).

Four dinucleotide Y chromosome microsatellite markers (*MS41A*, *MS41B*, *MS34A*, *MS34B*) were PCR amplified in 70 male coyotes (34 from Texas and 36 from Nebraska), 5 red wolves and 16 Mexican wolves, as described in [29]. A subset of samples was genotyped using newly designed primers (marked with the suffix -m). The original forward primers MS41a 5′-TCC TCT AAT TTT CCC CTC TA-3′ and MS41b 5′-TCC TCT AAT TTT CCC CTC TC-3′ from [29] were used with the new reverse primer MS41sR-m 5′-GAA GTC AGA CCC TTT ACC C-3′ to amplify the loci *MS41A* and *MS41B*. Loci *MS34A* and *MS34B* were amplified using the new primers MS34a-m 5′- ATA CAT TGC TGG ACG AGT GG -3′, MS34b-m 5′-ATA CAT TGC TGG ACG AGT CC-3′ and MS34sR-m 5′-TGA TTG GTG AAT GTC AAC ACA TGG ATG C-3′. These new primers were designed to amplify shorter DNA fragments and carry some deliberately introduced nucleotide mismatches compared to the original dog Y chromosome sequence [29], [35] to circumvent the formation of secondary structures by the primers. Resulting fragment sizes are 120 bp (*MS41A* and *MS41B*) and 63 bp (*MS34A* and *MS34B*) shorter than those from the original primers.

PCR reactions using the newly designed primers were performed in 10 µl two-loci multiplex reactions, one each for the *MS41* and *MS34* loci, containing 1× PCR buffer (Qiagen, Hilden, Germany), 2.7 mM (for loci *MS34A* and *MS34B*) or 3.2 mM MgCl_2_ (*MS41A* and *MS41B*), 0.3 mM of each dNTP, 0.4 µM of each of the two forward primers, 0.8 µM of the reverse primer, 0.025× Q solution (Qiagen), 0.04 U HotStar Taq polymerase (Qiagen) and approximately 10 ng of DNA template. PCR conditions were 15 min at 95°C followed by 38 cycles of 30 s at 95°C, 30 s at 61°C and 60 s at 72°C, and a final step of 10 min at 72°C. PCR products were diluted with water, mixed with ET-Rox 400 size marker (GE Healthcare, Uppsala, Sweden), and run on a MegaBACE 1000 instrument (GE Healthcare) according to the manufacturer's recommendations. Allele sizes were scored with the software provided with the instrument, Genetic Profiler 2.2.

### Data analyses

A neighbor-joining (NJ) phylogeny based on partial mtDNA control region sequences 393–400 base pair (bp) long (variation due to indels) was constructed in PAUP* 4.0b10 [36] using the HKY85 model of sequence evolution and a gamma correction (α = 0.5). Support for internal nodes was determined by 1000 bootstrap replicates. Haplotype and nucleotide diversity were calculated in DnaSP 4.50.3 [37].

The genotypes of the four Y chromosome microsatellites were combined into haplotypes because they are inherited as a single unit [29]. Haplotype diversity was calculated in Arlequin 3.11 [38]. Reconstruction of the phylogenetic relationships among these haplotypes requires a model of evolution. Given that most mutations within microsatellites result in changes of one repeat unit [39], [40], we calculated the number of mutational steps (addition or loss of a single dinucleotide repeat unit) for all pairwise comparisons of haplotypes, using a macro in Microsoft Excel™. Based on this distance matrix, a statistical parsimony network was constructed using TCS 1.21 [41].

## Results

### New Y chromosome microsatellite primers

We found the four Y chromosome microsatellite loci from [29] to be easier to amplify and less sensitive to PCR conditions when using the modified primers presented here. These features make the loci better suited for amplification in samples of suboptimal DNA quality and/or quantity, such as feces and historic museum material. The new primer sets may be particularly useful for management of the reintroduced population of red wolves in North Carolina, where coyotes are being excluded and red wolf–coyote hybrids are identified through noninvasive genetic surveys. Application of Y chromosome markers would facilitate the identification of hybrids resulting from the mating of female red wolves with male coyotes.

### Comparison of diversity levels

A total of 59 coyote mtDNA haplotypes were identified, 26 in the 53 coyotes from Texas, and 36 in the 71 coyotes from Nebraska (three were shared; Table 1, Table 2). The Texas coyote haplotypes differed by 1–24 substitutions (on average 8.0±1.3 SD among individuals) and contained six variable indels. The Nebraska coyote haplotypes differed by 1–24 substitutions (average 8.8±1.4 among individuals). Mitochondrial DNA haplotype and nucleotide diversity were similar in coyotes from Texas (π = 0.020±0.002) and Nebraska (π = 0.020±0.002) (Table 1).

**Table 1:** Genetic variability at mtDNA and Y chromosome microsatellite genotypes.

Species	population	mtDNA		Y chromosome	
		*N*_H_ (*n*)	*Hd*±SD	*N*_H_ (*n*)	*Hd*±SD
*Coyote*	Texas	26 (53)	0.949±0.016	15 (34)	0.920±0.025
	Nebraska	36 (71)	0.969±0.008	14 (36)	0.903±0.028
*Mexican wolf*	captive#	1 (6)	0	2	n.d.
	historic*	3 (6)	n.d.	n.d.	n.d.
*Red wolf*	captive#	1	0	2	n.d.

# from [18].

* from [26]. USNM 3188 and 3191 were labeled *C. l. baileyi* in previous study, but are excluded here as they have since been identified as *C. l. nubilus*, which leaves three haplotypes found in *C. l. baileyi* from USNM 15278, 95752, 98311, 98313, 58393 and 224484.

n.d. not determined.

*N*_H_ (*n*) denotes the number of unique haplotypes (*N*_H_) encountered in *n* individuals, and *Hd* is Nei's unbiased gene diversity [57].

**Table 2:** Occurrence of mtDNA control region haplotypes in coyotes from Texas and Nebraska.

Texas (*n* = 53)		Nebraska (*n* = 71)	
Haplotype	count	Haplotype	count
la006	9	la011*	1
la008	1	la012	7
la011*	2	la017	2
la027*	3	la021	2
la035*	1	la023	2
la054	2	la025	3
la086	3	la026	3
la087	1	la027*	6
la111	2	la028	3
la131	2	la030	1
la132	2	la031	2
la133	5	la032	1
la134	1	la033	3
la135	1	la034	5
la136	1	la035*	1
la137	2	la036	1
la138	1	la037	4
la139	1	la038	1
la140	2	la039	1
la141	4	la040	1
la142	1	la041	2
la143	1	la042	1
la144	2	la044	1
la145	1	la045	1
la146	1	la046	1
la147	1	la047	2
		la048	1
		la049	1
		la050	1
		la052	4
		la075	1
		la076	1
		la123	1
		la125	1
		la127	1
		la128	1

* Haplotypes shared among the populations.

Newly identified sequences have been submitted to EMBL, accession numbers FM209365-FM209425.

A total of 26 coyote Y chromosome haplotypes were identified, 15 in 34 coyotes from Texas, and 14 in 36 coyotes from Nebraska (three haplotypes were shared; Table 3). Y chromosome haplotype diversity was also similar in the two populations (Table 1; H = 0.920±0.025 in Texas; H = 0.903±0.028 in Nebraska). Overall, the coyote haplotypes differed from one another by 1–12 (5.1±2.3) mutational steps. Texas coyote haplotypes differed on average by 5.6±2.3 steps, and Nebraska coyote haplotypes by 4.2±2.1. For comparison, 20 haplotypes in 226 Alaska, United States and Northwest Territories (NWT), Canada gray wolves differed by 1–10 (average 4.6±2.3) mutational steps (data from [30], [31]).

**Table 3:** Details of Y chromosome haplotypes as defined by four microsatellites.

Haplotype	*MS41A*	*MS41B*	*MS34A*	*MS34B*	total frequency	occurrence
*H1*	208	218	174	178	1	RU
*H2*	208	214	176	178	1	TX
*H3*	212	220	172	178	2	NE
*H4*	212	222	172	178	2	NE(1), TX(1)
*H5*	212	214	172	180	2	NE
*H6*	212	216	172	180	3	TX
*H7*	212	218	172	180	3	NE
*H8*	212	220	174	174	4	TX
*H9*	212	214	174	176	1	TX
*H10*	212	220	174	176	5	TX
*H11*	212	224	174	176	7	TX
*H12*	212	226	174	176	1	TX
*H13*	212	214	174	180	1	TX
*H14*	212	210	176	178	3	NE(1), TX(2)
*H15*	212	212	176	178	6	RU(4), TX(2)
*H16*	212	220	176	178	1	TX
*H17*	212	222	176	178	1	NE
*H18*	212	220	178	176	3	TX
*H19*	214	212	172	178	1	NE
*H20*	214	214	172	178	8	NE
*H21*	214	216	172	178	3	NE
*H22*	214	218	172	178	7	NE
*H23*	214	220	172	178	2	NE
*H24*	214	224	172	178	2	NE(1), TX(1)
*H25*	214	216	174	178	1	NE
*H26*	216	210	172	178	3	NE
*H27*	218	214	172	176	1	TX
*H28*	208	218	172	178	6	MX
*H29*	208	220	174	178	10	MX
*H30*	208	214	172	176	33	AK(1)^a^, NWT(32)^b^
*H31*	208	226	172	176	9	NWT(1+8)^a^^,^ b
*H32*	208	214	172	178	26	AK(3)^a^, NWT(2+21)^a^^,^ b
*H33*	208	216	172	178	21	AK(3)^a^, NWT(18)^b^
*H34*	208	220	172	178	25	NWT(6+19)^a^^,^ b
*H35*	208	224	172	178	34	NWT(2+32)^a^^,^ b
*H36*	208	226	172	178	22	AK(2)^a^, NWT(20)^b^
*H37*	208	214	172	180	2	AK^a^
*H38*	208	222	172	180	29	NWT(1+28)^a^^,^ b
*H39*	208	220	176	178	2	AK(1)^a^, NWT(1)^b^
*H40*	208	218	178	176	1	NWT^a^
*H41*	208	212	172	178	2	NWT(2)^b^
*H44*	208	214	176	176	1	NWT^b^
*H45*	208	216	172	176	1	NWT^b^
*H50*	208	222	172	178	17	NWT^b^
*H52*	208	222	176	178	5	NWT^b^
*H53*	208	222	176	180	1	NWT^b^
*H55*	208	224	172	180	1	NWT^b^
*H58*	208	226	172	180	2	NWT^b^
*H59*	208	228	172	178	1	NWT^b^

a data from [30].

b data from [31].

Paternal lineages in coyotes from Texas (TX) and Nebraska (NE), captive red wolves (RU), Mexican wolves (MX), and gray wolves from Denali (Alaska, AK) and the Northwest Territories (NWT, Canada). Allele sizes are given as in [29].

In summary, comparison of variability levels at maternally and paternally inherited markers suggested that the genetic diversity of coyotes from Texas has not been dramatically increased by introgression of genes from other species.

### Introgression of female lineages

**Figure 2:**
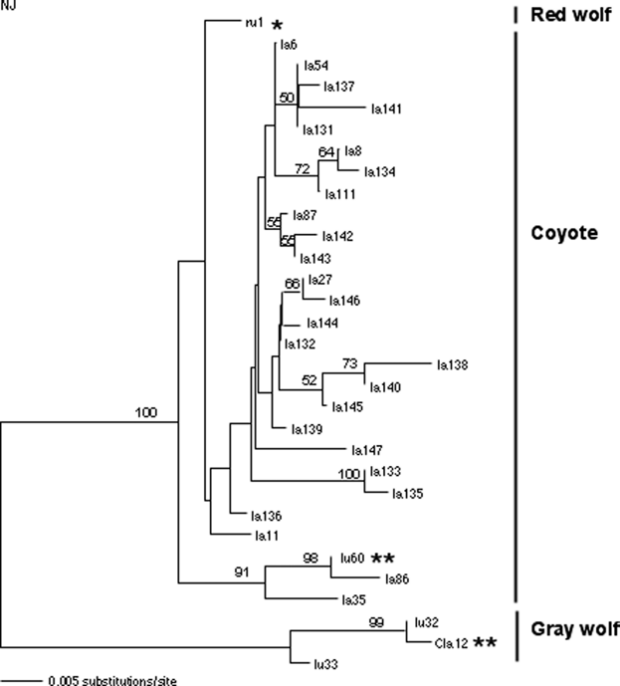
Phylogeny of mtDNA sequences. Neighbor-joining phylogeny of mtDNA control region sequences from coyotes from Texas (la), Mexican wolves (lu) and red wolves (ru). Bootstrap support is indicated on branches when over 50%. Single asterisk indicates possible hybrid origin, and double asterisks indicate haplotypes of clear hybrid origin.

All coyote mtDNA control sequences generated in our study (Table 2) formed a strongly supported monophyletic clade together with previously described coyote and captive red wolf sequences (Fig. 2). Only Texas coyotes are shown in the figure for clarity, but all haplotypes from Nebraska coyotes clustered with them (data not shown) see [17], [18], [26]. Further, all Texas and Nebraska coyotes analyzed here showed the indel pattern characteristic of the coyote mtDNA control region [17]. However, one Texas coyote previously analyzed by Adams *et al.* [13] had a haplotype (*Cla12*) located with high support in the gray wolf clade, most similar to haplotype *lu32*. Haplotype *lu32* is a relatively common gray wolf haplotype, widespread in North America [18], [26] and found in historic Mexican wolves (see below; [26]). This suggests that haplotype *Cla12* introgressed into the Texas coyote population following a mating between a male coyote and a female gray wolf (but see comments in the discussion regarding direct hybridization between gray wolves and coyotes). Overall, only one of more than 70 Texas coyote individuals studied in total has been found to carry gray wolf mtDNA, indicating limited introgression from the gray wolf lineage (Table 4; [13], [16], [18]; this study).

**Table 4:** Introgression in Texan *Canis* indicated by mtDNA or Y chromosome data.

Recipient taxon	Maternal lineages (mtDNA)	Paternal lineages (Y chromosome)
*Coyote*	gray wolf lineage introgressed [13] #	haplotype *H2* has allele 208 at locus *MS41A*, likely introgressed from red or Mexican wolves
*Mexican wolf*	coyote lineage introgressed into historic population [26] #	no introgression identified
*Red wolf*	original (historic) lineage unclear, but widespread introgression from gray wolf and coyote during decline [27], [28]	original, historic lineage unknown, but
		- *H1* carries 208 at *MS41A*, origin possibly red wolf or introgressed from Mexican wolf
		- *H15* may be introgressed from coyotes (is shared with Texas coyotes)

# Note that coyotes and gray wolves might not have been the ones that hybridized directly (see discussion).

A single mtDNA control region haplotype has been identified in captive Mexican wolves (haplotype *lu33*; [18]). This sequence is within the diversity of gray wolves, well separated from the coyote lineage [18], [26], and is not shared with any other gray wolf population studied to date. Consistent with this, evidence from other markers also does not suggest the presence of any hybrid lineages in the captive stock (reviewed in [42]). Three control region haplotypes have been identified in six historic Mexican wolves (Table 1) [26]. Haplotype *lu33*, found in the captive Mexican wolves, was also the most common among the historic sequences. The additional haplotypes found in historic Mexican wolves are *lu32* (a widespread gray wolf sequence, see above) and *lu60*, present in a single individual [26]. Haplotype *lu60* is closely related to a Texas coyote haplotype (*la86*; this study), from which it differs by two base changes, and groups with coyotes with high support (Figure 2, Table 4). This suggests that at some time in the past a female coyote hybridized with a male Mexican wolf, and their female offspring were incorporated into the Mexican wolf population. However, this mitochondrial lineage has not been found in the captive Mexican wolf population [18], [42].

The mtDNA control region haplotype found in captive red wolves (*ru1*; Fig. 2) was not identified in any Texas or Nebraska coyote (Table 2), although it clustered with them with high statistical certainty (Fig. 2). Haplotype *ru1* was most closely related to haplotype *la136* (found in a Texas coyote; this study), from which it differed by two substitutions (no indel).

Previously published mtDNA data from historic red wolf specimens showed both coyote-like and wolf-like haplotypes (3 of 6 gray wolf-like, 3 of 6 coyote-like, [27]; 3 of 11 gray wolf-like, 8 of 11 coyote-like, [28]). None of those historic sequences revealed a phylogenetically distinct lineage in red wolves, however this may be due to the lower resolution of cytochrome *b* sequences in *Canis*. The lack of reciprocal monophyly between known red wolf and coyote haplotypes makes phylogenetic conclusions regarding introgression considerably more difficult, but adds relevance to the above comparison of variation levels in coyotes from Texas and Nebraska. Phylogenetic analysis (Figure 2) shows that the captive red wolf haplotype falls within the diversity of coyote haplotypes, but that haplotype has not been found in any of the 86 Texan coyotes analyzed in this and other studies (Table 2) [13], [18], [43]. However, the large number of mtDNA haplotypes observed at low frequencies (Table 2) strongly suggests that many additional coyote haplotypes remain unsampled.

### Introgression of male lineages

**Figure 3:**
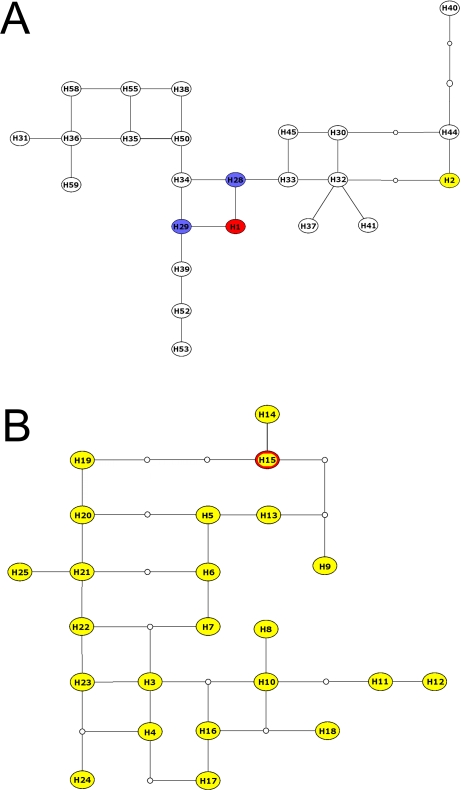
Statistical parsimony networks of Y chromosome haplotypes in North American *Canis*, based on four microsatellites. Coyote haplotypes are shown in yellow, Alaskan and Canadian gray wolves in white, Mexican wolves in blue, and red wolves in red. Inferred intermediate haplotypes are shown as small open circles. *A:* Haplotypes with the 208 allele at *MS41A*, characteristic of the gray wolf lineage. *H2* is a haplotype found in a Texas coyote with the 208 allele at locus *MS41A*. *B:* Haplotypes with alleles ≥212 at *MS41A*, characteristic of the coyote lineage. *H15* is shared between captive red wolves and coyotes from Texas.

With the exception of one coyote from Texas (haplotype *H2*), all male coyotes carried alleles of sizes 212–218 (with 212–214 found in >90% of individuals) at Y chromosome microsatellite locus *MS41A*. Previously published Y chromosome data from gray wolves report no alleles larger than 210. Allele 208 was identified in >98% of the more than 340 individuals analyzed to date (another variant, allele 210, was found in 5 wolves from the Baltic States and Russia) [29], [30], [31], [32]. Consequently, variation at locus *MS41A* appeared to be highly informative with regard to the wolf-coyote split. We used this locus to separate Y chromosome haplotypes into two groups, one of haplotypes showing the diagnostic gray wolf-like 208 allele at *MS41A*, and one of coyote-like haplotypes with alleles 212–218 (allele 210 has not been identified in any American wolf or any canid in this study). We show the evolutionary relationship between the haplotypes in the two groups separately (Fig. 3a and 3b). As mentioned above, one coyote from Texas had allele 208 at *MS41A* (haplotype *H2*), which indicates introgression of a non-coyote Y chromosome into the Texas coyote population (Table 4).

Two Y chromosome haplotypes were identified in 16 captive Mexican wolves (Table 3), likely reflecting the small number of founders. These two Mexican wolf haplotypes (*H28*, *H29*) carried the 208 allele at *MS41A*, characteristic of gray wolves, and differed from each other by two mutational steps (Fig. 3a). These haplotypes have not been identified in any other North American gray wolves analyzed to date [30], [31].

We identified two Y-chromosome haplotypes in five red wolves from the captive breeding program (Table 3). The two variants were relatively distantly related to one another. Haplotype *H1* had the wolf-like allele 208 at locus *MS41A* and was not identified in any other animal. This haplotype differed from Texas coyote haplotypes by 3–9 (5.6±1.5) mutational steps, and by only one step from each of the two Mexican wolf haplotypes (Fig. 3a). The second haplotype in the captive red wolf breeding program (*H15*) had the coyote-like allele 212 at locus *MS41A*, and was identified in two coyotes from Texas (one from Webb Co. and one from Andrews Co., 6% of the samples studied) (Fig. 3b). This haplotype differed from gray wolf haplotypes by an average of 8.0 steps (S.D. 2.6) and from coyote haplotypes by 1–9 steps (5.3±2.0 S.D.).

## Discussion

### Patterns of hybridization

Three morphologically well-separated species of *Canis* co-existed in Texas through the Holocene. During the 20^th^ century, however, widespread hybridization between red wolves and coyotes was reported [11], [12]. While it is possible that this process was historically ongoing at low frequency, extensive hybridization and introgression appear to be recent phenomena, likely resulting from anthropogenic habitat modification and dramatic population declines caused by direct persecution [12], [20].

We compared levels of genetic variability in coyotes from Texas, which were historically sympatric with Mexican wolves and red wolves, with that in coyotes from Nebraska, which were historically sympatric with gray wolves only. Even if the red wolf and coyote are too closely related to have reciprocally monophyletic mitochondrial lineages, extensive hybridization between them could have led to an increase in genetic variability in the remaining coyote population. However, our results from both maternally and paternally inherited markers did not show any strong evidence for elevated levels of variation in Texas coyotes. This suggests that introgression into coyotes was rare compared with the total size of the coyote population.

Phylogenetic analyses did reveal instances of hybridization, although an accurate assessment of the degree of introgression was difficult due to uncertainty in identifying endemic red wolf haplotypes. Size homoplasy in the Y chromosome microsatellites, which was suggested by multiple connections among haplotypes (Figure 3; see also [30]), could add further uncertainty. However, inspection of our data and published Y chromosome data revealed that all American gray wolves carry a diagnostic allele (208) at locus *MS41A*, while coyotes have alleles 212–218. Genetic differentiation at maternal and paternal markers thus allowed us to identify several lineages that had introgressed into another species. These data revealed that all three native *Canis* species from Texas had participated in hybridization events to some degree (see Table 4).

#### Abundance-related impact of introgression

The genetic signal of introgression was not equal in the different species. Hybridization events between red wolves and both Mexican wolves and coyotes appear to have resulted in introgression most often into the red wolf population. While the red wolf and coyote populations apparently accepted male and female hybrids, Mexican wolves only show evidence of accepting female hybrids. Altogether, this may illustrate the critical situation of the red wolf population as it was going extinct in the wild, with density-dependent (Allee) effects leading to relatively high introgression rates into red wolves. Differences in mating preferences and/ or breeding periods may also have contributed to this pattern.

Only two Texas coyotes studied so far appear to carry introgressed alleles-a single coyote with a gray wolf-like mtDNA haplotype, and a single coyote with the gray wolf-like *H2* Y chromosome haplotype. Available data therefore suggest that Texas coyotes have withstood the last centuries' ecological changes without much introgression from sympatric species of *Canis*, with which they have been documented to hybridize ([11]; Table 4). Coyotes have been common and widespread in Texas throughout historic times, so backcrossing of red wolf–coyote hybrids into the coyote population could be regarded as unlikely under the “*scarcity of mates*” hypothesis (see [1], [7]). Additionally, such backcrossing to coyotes may be expected to have left only a minor genetic footprint, given the large population size of Texas coyotes.

#### Sex and size-related biases in hybridization patterns

Allee effects may affect the sexes differently, and it has been suggested that hybridization between canids should involve a male of the larger species and a female of the smaller species [15]. Indeed, the presence of a wolf-like *H2* Y chromosome in Texan coyotes indicates mating between a female coyote and a larger male wolf, as does the presence of coyote mtDNA in a historic Mexican wolf. However, we also found evidence of the opposite pattern. Evidence of smaller male coyotes mating with larger female wolves include the presence of gray wolf mtDNA in a Texas coyote and the coyote-like Y chromosome haplotype *H15* in red wolves. These data show that female as well as male coyotes were involved in hybrid matings, which implies that both sexes mated with larger partners. In summary, neither sex nor size bias hypotheses alone can explain all of the data.

Although coyotes and gray wolves are known to produce fertile offspring in captivity [44], hybridization appears to have occurred only very rarely across their extensive zone of overlap in North America [15]–[18]. Perhaps the presence of the intermediate-size red wolf was an important factor in breaking down reproductive barriers and leading to this *ménage-à-trois*. The medium-sized red wolf could have hybridized with both the smaller coyote and the larger Mexican wolf, and in doing so transmitted genetic material of hybrid origin. In this context it is noteworthy that the second zone of extensive introgression between coyotes and gray wolves is in the Great Lakes area, where another intermediate-size wolf occurs [14], [43].

### Captive populations

Both the captive population of red wolves and the captive population of Mexican wolves show low levels of genetic diversity, which is to be expected given the severe bottleneck imposed by limited numbers of founders and subsequent captive breeding (in total 7 founders for the three lineages involved in the Mexican wolf captive breeding program, 14 founders for the captive red wolves [45]). Evidence of introgression of a coyote mitochondrial haplotype was identified in a historic Mexican wolf, but this lineage is not present in the extant population. None of the maternally or paternally inherited lineages in the Mexican wolf captive breeding program appear to have a hybrid origin (some introgressed nuclear genes could remain, but see [46] who found evidence for purity of the captive stock at autosomal microsatellite markers).

The situation for the red wolf captive breeding program is different, as both the mitochondrial and both Y chromosome lineages could have a hybrid origin. However, this is more difficult to determine accurately, because pre-decline haplotypes are not known for these markers. Unfortunately, genetic variation on the Y chromosome is very limited in mammals [47], hampering the analysis of single nucleotide polymorphisms that could clarify the phylogeny of Y chromosome haplotypes.

The taxonomic origin of the captive red wolf mtDNA haplotype (*ru1*) is uncertain. It is thought that red wolves are closely related to coyotes [20], and therefore it is possible that red wolves and coyotes are not reciprocally monophyletic due to incomplete lineage sorting [48], [49]. Alternatively, haplotype *ru1* may actually be of coyote descent. If so, this variant may have entered the red wolf population through introgressive hybridization with coyotes when the red wolf was going extinct in the wild (Table 4).

One of the Y chromosome haplotypes found in red wolves fell within the genetic diversity of coyotes (haplotype *H15*), and the other (*H1*) within the diversity of gray wolves (Fig. 3). Haplotype *H15* was also found in two extant coyotes from Texas, indicating that it may have been introgressed from coyotes into red wolves (or vice versa). Similarly, the phylogenetic proximity of the second (wolf-like) captive red wolf Y chromosome haplotype *H1* to the two found in Mexican wolves could indicate that it introgressed into the red wolf population, or that *H1* represents an authentic red wolf lineage that is similar to the Mexican wolf haplotypes at the studied Y chromosome microsatellites (Table 4).

### Implications for reintroductions

Reintroduced Mexican wolves have not been threatened by hybridization, although the potential for them to hybridize with domestic dogs and coyotes does exist. If the reintroduced population of Mexican wolves is to be self-sustaining, its population size will have to increase. If the population remains too small, individuals will not be able to find another unrelated Mexican wolf for a mate. If individuals are unable to find a suitable mate, they may be susceptible to mating with individuals of other species or may forgo breeding altogether (*i.e.* [50]).

While the phylogenetic origin of maternally and paternally inherited genetic markers in the captive red wolf program remains unclear, captive animals appear similar to historic red wolves in morphology [20] and autosomal microsatellites [51]. Signs of introgression at mitochondrial markers despite apparent purity in the nuclear genome have been found in other mammals (*e.g.* African elephants [52], goats [53] and chipmunks [54]). Ongoing attempts to reintroduce the red wolf into the wild should therefore not be affected by the presence of introgressed haplotypes. Reintroduction of the red wolves is important because they fill an important ecological niche that was left empty with their eradication.

Although red wolf–coyote hybridization apparently did not have a major impact on the Texas coyote population, it had [12] and continues to have a major impact on the red wolf population [13], [55]. Hybridization with Mexican wolves may have had an important impact on the red wolf population historically. However, this is no longer a threat to the red wolf now that the species are completely allopatric.
